# Sugar-Sweetened Beverage Consumption and Its Association With Dental Caries Among Adolescents in Erbil, Iraq: A Cross-Sectional Study

**DOI:** 10.7759/cureus.58471

**Published:** 2024-04-17

**Authors:** Heran I Hassan, Samir M Othman

**Affiliations:** 1 Orthodontics, Pediatric and Preventive Dentistry, College of Dentistry, Hawler Medical University, Erbil, IRQ; 2 Community Medicine, College of Medicine, Hawler Medical University, Erbil, IRQ

**Keywords:** public health, common risk factor approach, oral health, 24h recalls, adolescences, dental caries, sugar-sweetened beverages

## Abstract

Background

Sugar-sweetened beverages are one of the most common sources of added sugar in the diet and have been associated with an increased risk of dental caries, obesity, major chronic diseases, and possibly cancer. Dental caries is a diet-related, highly prevalent, and preventable oral disease. The objective of this study was to assess the frequency of sugar-sweetened beverage consumption and its association with dental caries in adolescents in Erbil, Iraq.

Methods

This is a cross-sectional study that invited 11- to 16-year-old intermediate school students (n=380) in Erbil, Iraq. Data collection comprised a validated questionnaire and a clinical examination. In addition, dietary data were collected by using two non-consecutive 24-hour recalls. The mean of the daily consumption of sugar-sweetened beverages over a two-day period was calculated. Dental caries was diagnosed based on World Health Organization (WHO) criteria and was reported as a decayed, missing, and filled permanent tooth (DMFT). A multiple regression model was used to assess the influence of sugar-sweetened beverages on dental caries experience (DMFT). SPSS version 26 (Armonk, NY: IBM Corp) was used to analyze the data at the 5% significance level.

Results

Of the 380 students interviewed, one participant was excluded because of incomplete data. The participant's mean age and standard deviation (SD) were 13.3±1.2. A total of 54.1% of the students were female. The mean daily intake of sugar-sweetened beverages was 686.71±197.50 mL per day. Male students consumed more beverages than female students (p<0.001). The most frequently consumed sugar-sweetened beverages were sweetened tea and coffee, and the least frequently consumed beverages were milk and dairy products. The mean decayed, missing, and filled permanent tooth (DMFT) was 4.58±2.73. Results of multiple regression analysis showed that caries experience (DMFT) was associated with insufficient toothbrushing (p<0.001), plaque-affected sextants (p=0.001), and male sex (p=0.016). The model also showed a significant association between sugar-sweetened beverage consumption and dental caries experience (DMFT) (regression coefficient=0.008, CI: 0.006-0.009, p<0.001).

Conclusions

Adolescents in Erbil, Iraq, consumed sugar-sweetened beverages on a frequent basis, and male students consumed more sugary beverages than females. The higher frequency of these beverage consumptions was associated with a higher dental caries experience. Consequently, reducing sugar-sweetened beverage consumption could have a significant positive public health impact.

## Introduction

Global consumption of sugar-sweetened beverages (SSBs) is increasing in low- and middle-income countries, and in many high-income countries, intake levels are beyond the daily recommended limits for free sugar [1]. The reduction of free sugar consumption to <10% of total energy intake throughout all life phases is advised by the World Health Organization (WHO), as a means to mitigate the potential hazards of unintended weight gain and dental caries [2]. According to estimates, the intake of SSBs worldwide is linked to around 184,000 deaths annually. Of these, over 133,000 deaths are attributed to diabetes mellitus (DM), 45,000 deaths to cardiovascular diseases (CVD), and 6,450 deaths to cancer. Among countries with varying income levels, 5.0% of SSB-related deaths happened in low-income countries, 70.9% in middle-income countries, and 24.1% in high-income countries [3]. Consumption of SSBs exhibits significant variations based on geographic region, gender, age, and socioeconomic position. This consumption is often higher among younger individuals, boys, and low-income groups [3]. The consumption of dietary sugar and SSBs among children and adolescents in the United States surged notably in the years preceding 2000, followed by a sharp and sustained decline. Unlike the fluctuating trends observed in the United States, global consumption patterns of SSBs and dietary sugars remained relatively stable or exhibited slight variations over a span of three decades [4]. A study conducted in Duhok, Iraq, focusing on adolescents aged 15-24 years, reported that 24.8% of participants ingest a minimum of four "istican" (a local measurement equivalent to 30cc) of tea daily, while 70.8% consume four or more soft drink cans per week [5].

Sugar-sweetened beverages are known to be significant risk factors for negative oral health outcomes due to their high sugar content and acidity. As a result, they contribute to the formation of dental caries and tooth erosion [6,7]. According to an extensive systematic review, moderate-quality evidence suggests that consuming <10% free sugar reduces dental caries. Dental caries progression is often exacerbated with age, and the detrimental effects of sugar on dental health are enduring. Apparently, low levels of tooth decay in childhood have a significant impact on the occurrence of tooth decay throughout a person's life. Hence, regulating sugar intake to less than 5% of total energy (E) holds promise for mitigating the risk of dental caries across various stages of life [7]. Dental caries is a result of acids produced in the mouth by microorganisms metabolizing sugar, which is the widely accepted mechanism underlying the association between SSB consumption and dental caries. Increased sugar content in SSBs increases acid production and enhances additional tooth damage [8]. Dental caries in the permanent dentition that is not treated is the most common condition among all human illnesses, impacting almost 2.3 billion individuals worldwide [9,10]. An assessment of dental caries at a regional level revealed significant social inequality, with the impoverished and marginalized population being significantly more affected [11]. The Global Burden of Disease (GBD) research has regularly shown that oral health is a significant worldwide public health concern that has been ignored [10,12]. The GBD 2015 [13] data were used to support preventing oral problems as part of efforts to stop non-communicable diseases (NCDs) and make dental care available to everyone [14,15]. Continual national and worldwide evidence on the descriptive epidemiology of common oral conditions is necessary for government and non-governmental organizations [9]. An integrated approach that combines efforts to promote oral health and address unhealthy behaviors has the potential to decrease dental diseases and mortality rates associated with cardiovascular disease, cancer, and diabetes. This approach is not only more cost-effective but also prevents the duplication of efforts or the contradiction of health messages [16].

Studies acknowledge adolescence as a crucial stage of life when it comes to habits related to food intake. During this phase, adolescents allocate a greater amount of time to socializing with their peers and also exercise greater autonomy in making dietary decisions [17]. Frequently engaging in unhealthy eating habits, such as avoiding nutritious meals and consuming sugary beverages, might have detrimental effects on health during this time [18]. Given that numerous lifestyle behaviors are formed during this specific period [19] and young adults and adolescents constitute the highest consumers of SSBs on a global scale, this study is conducted among adolescents in Erbil to assess their frequency of SSB consumption and its effect on dental caries experience among this important age group [3,20].

To the researcher’s best knowledge, there was no study to specifically evaluate the frequency of SSB consumption and its effects on the dental caries experience in this region. By focusing on local data, this research may provide relevant information that can inform targeted interventions and policies tailored to this area's needs. This research can serve as a foundation for future studies investigating the effectiveness of interventions aimed at reducing SSB consumption and preventing dental caries. Accordingly, the aim of this study was to assess beverage consumption frequencies and to evaluate their contribution to dental caries experiences among adolescents in Erbil.

## Materials and methods

Research design and subjects

This cross-sectional study was carried out during the scholastic year 2022-2023, from October 2022 to April 2023, and targeted both male and female students between the ages of 11 and 16 years, intermediate school children attending public schools in Erbil, Kurdistan Regional Government (KRG), Iraq. Erbil is divided into six municipal zones according to managerial factors. Out of 250 schools, 14 schools were randomly selected according to the size of each zone. The sample size was calculated using Epi Info version 7 (Atlanta, GA: Centers for Disease Control and Prevention) using the following parameters: total population=27,545, confidence interval (CI)=95%, expected frequency=50%, and margin of error=5%. The estimated sample size was n=380. The probability proportional to the size sampling technique was used to select the schools. Within each school, 27-30 students were randomly selected from grades 7, 8, and 9 according to the distribution of the students across each stage. Parents were informed in writing about the purpose of the study and were invited to contact the researchers or respective schools for any inquiries or to withdraw their child from the study. The study's aims and methodology were explained, and signed informed consent was obtained from the parents and the students prior to data collection in accordance with the principles outlined in the Helsinki Declaration. Confidentiality was approved. The eligibility criteria included students aged 11 to 16 years who were free from any systemic disease, not taking medications, and not wearing orthodontic appliances. The Research Ethics Committee of the College of Dentistry, Hawler Medical University approved the study proposal on July 13, 2021 (meeting code 4, paper code 10).

Sociodemographic characteristics

Questions about age, gender, grade, parental education, parental occupation, the Family Affluence Scale, systemic disease, medication, and oral health-related habits (tooth brushing frequency, use of fluoride toothpaste, and dental visits) were collected from the students. The vast majority of the questions in the questionnaire were derived from the validated health-related behavior in school-aged children survey protocol [21].

Portion size estimation

In this survey, two validated 24-hour recall methods were adopted [22]. In this study, a Photographic Atlas of Food Portion for Abu Dhabi and photographs of local foods prepared by local nutritionists were used for portion size calculation [23]. In addition, commonly used glasses and cups in this population were coded and used to help those participants who couldn’t find matched cups and glasses in the atlas. Then data were entered into myfood24, which is an online dietary assessment tool developed by the University of Leeds in the United Kingdom [24]. Unfortunately, the lack of access to the internet and the cognitive capacity of the sample, as well as the language barrier, made the use of myfood24 directly by the participants difficult. The data were collected by the researchers and entered into the myfood24 software by the researchers (HH).

In order to facilitate a clear comprehension of the findings, the portion of 250 mL per day was used to measure the frequency of consumption. The frequencies of beverage consumption were categorized into four distinct groups as follows: <2 portions per day, 2-2.9 portions per day, 3-3.9 portions per day, and ≥4 portions per day. Data pertaining to eight drinks, namely milk, natural fruit juice, tea and coffee beverages, soft beverages, and artificial fruit juice, were documented. These drinks were categorized into five classes. The first group was labeled as “hot beverage” which included black tea, coffee, and Nescafe, the second carbonated drink category consisted of soft drinks. The third item on the list was "fruit juice," the fourth item was “energy drinks” which included caffeinated beverages, while the fifth item was "milk and dairy products," which included sweetened whole milk, chocolate milk, and milkshakes.

The Family Affluence Scale (FAS) III comprises six items that assess the family's material wealth, the FAS was used in this research as both a continuous variable ranging from 0 to 13 and also categorized into the following cut-offs: 0-7 (low), 8-11 (mid), and 12-13 (high) levels of family wealth [25]. Parental education is classified as follows: illiterate/can read and write, primary, secondary, institute, college and above. Parental occupation is classified as follows: high rank, non-manual worker, skilled manual worker, unskilled/unemployed. We considered FAS as a measure of wealth and it is combined with parental education and parental occupation to calculate socioeconomic status (SES) [25].

Tooth brushing

We assessed tooth brushing practices with the following query: "How frequently do you engage in tooth brushing? The response options were as follows: many times daily, daily, at least weekly but not daily, less than weekly, never. Tooth brushing was dichotomized as less than once a day and at least once a day [26]. Fluoride exposure was measured by asking the following query: does toothpaste have fluoride? The response options were as follows: yes, no, I don’t know [27]. Visiting a dentist in the last six months was binary (yes or no). The visit to the dentist was recorded by the question “Have you visited a dentist in the last six months?” and the possible answers were “yes” or “no” [28]. Furthermore, the degree of plaque was visually assessed using the Plaque Index (PlI), and binary conclusions were made for each sextant about the existence of dental plaque [29]. The total number of plaque-affected sextants ranged from 0 to 6 [30].

Clinical oral examination

The clinical oral examination was carried out by a trained and calibrated dentist according to WHO criteria, and the examiner had two sessions of training including one calibration session (Kapa 80%) [31]. All participants had examinations while seated in a comfortable position, using a halogen light, a plane mirror, and the Community Periodontal Index (CPI) probe. Moisture management was achieved by using cotton rolls. The decayed, missing, filled surfaces (DMF/S) index was used to quantify caries experience at both the surface and tooth level. A decayed tooth was recorded in the presence of pit and fissure and or smooth surface lesions with softened floor, undermined enamel, or softened wall. Temporary fillings are considered decayed. Proximal surface lesions were recorded when the probe entered the lesion with certainty. On the other hand, stained pits and fissures without undermined enamel and softened walls and floors were not recorded as being carious. A tooth with permanent restorations was recorded as filled. Only missing teeth due to caries like those with a history of pain or cavitated teeth prior to extraction were recorded as missed teeth [32].

Statistical analysis

The data analysis was conducted using the Statistical Package for the SPSS version 26.0 (Armonk, NY: IBM Corp.). Descriptive statistics were employed to determine the frequencies and percentages of categorical variables, along with the means and standard deviations of continuous numerical variables. Assessment of the normality of decayed, missing, and filled permanent tooth (DMFT) data was performed using the Shapiro-Wilk test, revealing a non-normal distribution (p<0.001). Consequently, non-parametric tests were utilized. The Spearman's correlation test was employed to explore the relationships between DMFT and both total sugar and total SSBs sugar. The Mann-Whitney test was applied for comparing means between two groups, while the Kruskal-Wallis test was used for comparisons involving three or more groups. In the multivariate regression model, control was exercised for variables under investigation and those previously identified with significant associations with dental caries. Statistical significance was defined as a p≤0.05.

## Results

The total number of participants interviewed was 380, one student was excluded due to incomplete data. The participants' mean age and standard deviation (SD) were 13.3±1.2. The age range was 11-16 years. About 43% of the sample fell within the age group of 13-14 years, and 54% were female. In terms of parental education, fathers were either institute graduates (27%) or college graduates (29%), compared to 11% and 20% of the mothers, respectively. Sixty-three percent of fathers were employed in skilled manual occupations, while 33% were engaged in non-manual occupations. Conversely, 65% of the mothers were identified as housewives. Regarding socioeconomic status (SES), 45% of the students belonged to medium SES (Table 1).

**Table 1:** Participants' basic characteristics.

Variable	n	%
Age (years)		
11-12	126	33.2
13-14	162	42.7
15-16	91	24.0
Gender		
Male	174	45.9
Female	205	54.1
Father education		
Illiterate/read and write	4	1.1
Primary	79	20.8
Secondary	87	23.0
Institute	101	26.6
College and above	108	28.5
Mother education		
Illiterate/can read and write	48	12.7
Primary	109	28.8
Secondary	103	27.2
Institute	77	20.3
College and above	42	11.1
Father occupation		
High rank	11	2.9
Non-manual worker	123	32.5
Skilled manual workers	239	63.1
Unskilled and unemployed	6	1.6
Mother occupation		
High rank	2	0.5
Non-manual workers	129	34.0
Unskilled manual	2	0.5
Housewives	246	65.0
Socioeconomic status		
Low	140	36.9
Medium	169	44.6
High	70	18.5
Total	379	100.0

Regarding the Family Affluence Scale, only 14% of the students reported not having a family car, and the rest either had one car (67%) or two cars (19%). About 41% of the students had their own bedroom, and 58% had one computer. Sixty-one percent of the sample had one bathroom, and 27% had a dishwasher. Moreover, 45% of the students never traveled abroad (Table 2).

**Table 2:** The Family Affluence Scale.

Items	n	%
Car ownership		
No	52	13.7
Yes, one car	254	67.0
Yes, two or more cars	73	19.3
Own bedroom		
No	224	59.1
Yes	155	40.9
Computer ownership		
None	75	19.8
One	221	58.3
Two	60	15.8
More than two	23	6.1
Number of bathrooms		
None	0	0.0
One	232	61.2
Two	105	27.7
More than two	42	11.1
Dishwasher		
No	276	72.8
Yes	103	27.2
Travel outside		
Not at all	172	45.4
Once	78	20.6
Twice	79	20.8
More than twice	50	13.2
Total	379	100.0

Distributions of the participants' oral-health-related habits showed that 19% of the students used to brush their teeth more than once a day and 44% reported brushing their teeth once a day. Seven percent of the respondents reported never brushing their teeth. Seventy-three percent mentioned that they used fluoride toothpaste (Table 3).

**Table 3:** Participants' oral-health-related practices.

Variable	n	%
Toothbrush		
More than once a day	70	18.5
Once a day	167	44.1
At least once a day but not daily	73	19.3
Less than once a week	41	10.8
Never brush	28	7.4
Fluoride		
No	28	7.4
Yes	277	73.1
Don’t know	74	19.5
Total	379	100.0

The pattern of consumption of various sugar-sweetened beverages among the study participants was as follows: hot drinks (tea and coffee) were the most frequently consumed beverages by 82% of the participants. Carbonated drinks were consumed by 68% followed by fruit juice 50% and then energy drinks 29%. The least commonly consumed beverages were milk and dairy products 17 (Table 4).

**Table 4:** Patterns of SSB consumptions. SSB: sugar-sweetened beverage

Type of drink	No.	% (n=378)	Mean (mL/day)	Median	Min.	Max.
Hot drinks	310	82.0	229.0	190.0	80.0	790.0
Carbonated drinks	255	67.5	350.9	250.0	150.0	1100.0
Fruit juice	189	50.0	268.1	200.0	150.0	800.0
Energy drinks	111	29.4	266.0	250.0	150.0	500.0
Milk and dairy products	65	17.2	194.5	200.0	100.0	450.0

**Figure 1:**
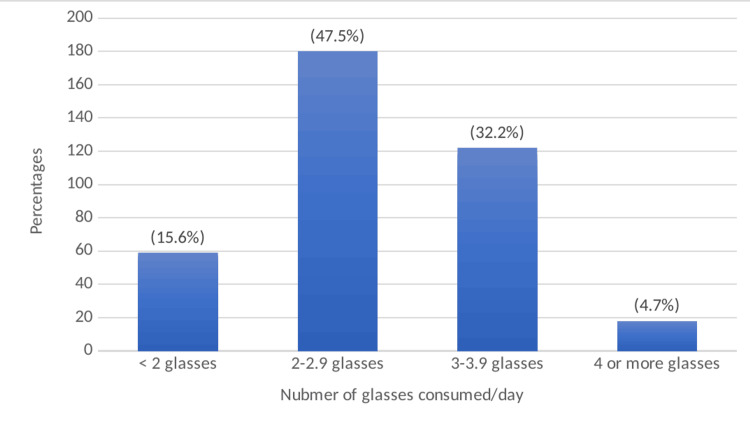
Frequencies of the participants' sugar-sweetened beverage consumption. One glass = one portion = 250 mL.

Figure 1 below shows the frequencies of intakes of sugar-sweetened beverages on a given day. Almost 48% of the students consumed 2-2.9 glasses (portions) of SSB per day, 32% of the students consumed 3-3.9 glasses, while 16% of the students consumed <2 glasses per day. Only 5% of the participants consumed four or more portions.

Positive significant correlations were detected between DMFT with the following variables: decayed, missed, and filled permanent tooth surfaces (DMFS) indicating a strong positive correlation (rho=0.881, p<0.001), total sugar showing a moderate positive correlation (rho=0.465, p<0.001), and total SSB sugar indicating a moderate positive correlation (rho=0.633, p<0.001). The correlations between DMFS and the mentioned variables were also positive and significant but weaker. More details are presented in Table 5.

**Table 5:** Correlations between dental indicators and age and sugar. DMFT: decayed, missing, and filled permanent tooth; DMFS: decayed, missed, and filled permanent tooth surfaces; rho: Spearman’s correlation coefficient

Variable 1	Variable 2	rho	p-Value
DMFT	DMFS	0.881	<0.001
DMFT	Total sugar in grams	0.465	<0.001
DMFT	Total SSB sugar	0.633	<0.001
DMFS	Total sugar in grams	0.398	<0.001
DMFS	Total SSB sugar	0.520	<0.001
Total sugar in grams	Total SSB sugar	0.653	<0.001

The more the intake of the SSB (the more cups of SSB per day), the more the means and mean ranks of the following oral health indicators: DMFT, DMFS, and plaque-affected sextants. All the differences were significant (p<0.001) as presented in Table 6.

**Table 6:** Associations between oral health indicators and SSB portions. *P-value calculated by using Kruskal-Wallis test. DMFT: decayed, missing, and filled permanent tooth; DMFS: decayed, missed, and filled permanent tooth surfaces; SSB: sugar-sweetened beverage

Dental indicators	Cups of SSB per day	n	Mean	SD	Mean rank	p-Value*
DMFT	<2	59	2.46	1.62	95.41	<0.001
	2-2.9	180	3.62	1.90	153.88	
	3-3.9	122	6.48	2.56	269.86	
	≥4	18	8.28	2.44	320.00	
	Total	379	4.58	2.73	-	-
DMFS	<2	59	4.54	2.89	110.24	<0.001
	2-2.9	180	6.33	3.52	159.62	
	3-3.9	122	10.11	4.29	254.75	
	≥4	18	14.00	5.11	316.42	
	Total	379	7.63	4.52	-	-
Plaque-affected sextants	<2	59	2.71	1.83	132.89	<0.001
	2-2.9	180	3.16	1.57	155.18	
	3-3.9	122	4.46	0.94	254.12	
	≥4	18	4.89	0.76	290.83	
	Total	379	3.59	1.59	-	-

The mean and mean rank of DMFT were significantly high 5.96 and 250.48, respectively, when the patients were not using fluoride toothpaste, compared with 4.41 and 182.95, respectively, when the patients were using fluoride toothpaste (p=0.007). The same can be applied to DMFS where the mean and mean rank of DMFT were significantly high when the patients were not using fluoride toothpaste (p=0.012). Regarding teeth brushing, the mean and mean rank of DMFT and DMFS among those who didn't brush their teeth sufficiently were significantly higher than those children who brushed their teeth sufficiently (p<0.001) (Table 7).

**Table 7:** Association between oral-health-related habits and dental caries experience. *P-value calculated by using Mann-Whitney U test. **P-value calculated by using Kruskal-Wallis test. DMFT: decayed, missing, and filled permanent tooth; DMFS: decayed, missed, and filled permanent tooth surfaces

Indicators	Variables	n	Mean	SD	p-Value
Tooth brushing					
DMFT	Sufficient	237	3.77	2.44	<0.001*
	Not sufficient	142	5.93	2.66	
DMFS	Sufficient	237	6.49	4.25	<0.001*
	Not sufficient	142	9.54	4.32	
Dental visits					
DMFT	Yes	144	4.51	2.62	0.863
	No	235	4.62	2.79	
DMFS	Yes	144	7.54	4.28	0.946
	No	235	7.69	4.67	
Fluoride toothpaste use					
DMFT	No	28	5.96	2.32	0.007**
	Yes	277	4.41	2.72	
	Don’t know	74	4.68	2.79	
DMFS	No	28	10.04	4.19	0.012**
	Yes	277	7.37	4.54	
	Don’t know	74	7.72	4.36	

Results of multiple regression analysis showed that DMFT scores were associated with average SSB (mL/day) (p<0.001), insufficient brushing (p<0.001), plaque-affected sextants (p=0.001), and male sex (p=0.016). Following adjustment for the confounding variables, the multiple regression model showed a significant association between DMFT and SSB (mL/day) (regression coefficient=0.008, CI: 0.006-0.009) (Table 8).

**Table 8:** SPSS output of multiple regression analysis between DMFT scores (as the dependent variables) and several covariates. DMFT: decayed, missed, and filled permanent tooth

Variables	Unstandardized coefficients		Standardized coefficients	t	p-Value	95.0% confidence interval for B	
	B	Std. error	B			Lower bound	Upper bound
Constant	-6.091	1.285	-	-4.738	<0.000	-8.618	-3.563
BMI Z score	-0.093	0.128	-0.033	-0.720	0.472	-0.345	0.160
Insufficient brushing	1.741	0.217	0.309	8.030	<0.001	1.315	2.168
SSB (mL/day)	0.008	0.001	0.518	9.435	<0.001	0.006	0.009
Total kcal	0.000	0.001	0.021	0.450	0.653	-0.001	0.001
Total sugar (g)	0.006	0.004	0.069	1.380	0.168	-0.003	0.015
Plaque-affected sextant	0.238	0.070	0.136	3.385	0.001	0.100	0.377
Male sex	0.515	0.212	0.094	2.429	0.016	0.098	0.932
Fluoride toothpaste	0.210	0.234	0.034	0.901	0.368	-0.249	0.670

## Discussion

This study was carried out among adolescents in Erbil in order to determine the frequency of SSB consumption and its effect on the dental caries experience. Adolescents are in a crucial stage of development where they are forming long-lasting health behaviors, including dietary habits. This age group is more likely to have autonomy in making dietary choices, including their beverage preferences. Studying SSB consumption during this period can provide insights into the establishment of dietary patterns and habits that may persist into adulthood. To the best of the researcher's knowledge, there was no study that particularly evaluated the frequency of SSB consumption and its impact on the dental caries experience among adolescents in this region. This research has the potential to establish a basis for subsequent studies that examine the effectiveness of interventions aimed at decreasing SSB intakes and preventing dental caries. This research may provide important information that can inform targeted interventions and policies by focusing on local data. Accordingly, the aim of this study was to determine the frequency of SSB consumption and evaluate its impact on dental caries experience among adolescents in Erbil.

The results of this study showed that the consumption of SSBs was relatively high among adolescents in Erbil, Iraq. Boys consumed significantly more SSBs than girls. Tea and coffee were the most commonly consumed beverages, while milk and dairy products were the least consumed beverages. The study reported a very high caries experience in this population. Following the control of confounding variables, the results of the multivariate regression modeling showed a significant association between SSB intake and dental caries experience (p<0.001).

Regarding SSB consumption, adolescents in Erbil consumed a relatively high amount of SSB; the average daily consumption was 2.7469 cups, or (mean±SD) 686.71±197.50 mL per day. Eighty-two percent drank hot beverages, whereas milk and dairy products made up the least amount of SSB consumption (17%). Boys consumed more SSB compared to girls, and this trend was in parallel with the results of a study conducted in Mosul city, Iraq. The study reported that the daily consumption of milk was decreasing among both boys and girls, with around one-third of them regularly consuming milk. Boys exhibited greater intakes of sugar-sweetened drinks and energy drinks compared to girls (p<0.01). The study highlighted poor dietary and lifestyle behaviors among adolescents, with significant gender differences in most behaviors. This suggested that the nutritional shift in Iraq, although it began later than in the nearby Arab Gulf countries, has rapidly caught up [33].

Adolescents face various influences that contribute to their consumption of energy-dense, low-nutrient foods and beverages. Factors at the intrapersonal level, such as nutritional knowledge, self-efficacy, and taste preferences, play a significant role in determining the consumption of SSBs and sugary snacks. Interpersonal influences, including parental and peer influences, also affect consumption patterns. Furthermore, environmental factors like the availability of these beverages at home, school, and neighborhoods close to their home contribute to shaping adolescents' dietary choices [34]. Many locally produced, cheap, and high-sugar-content beverages with little or no nutritional value are available to children and adolescents in Erbil. Out of the 14 schools included in this study, only one school for girls reported prohibiting the sales of carbonated beverages and energy drinks; however, fruit juice, milkshakes, tea, and coffee had not been prohibited in the school canteen. In addition, social media and television food commercials frequently promote the consumption of unhealthy foods and beverages, which has been linked to increased consumption among teens. Peer pressure and influencers increase the effects of marketing, which also interact with social, biological, and environmental variables [35]. A systematic review and meta-analysis presented the latest data on the consumption of SSBs by children and adolescents in 51 countries. The review highlights that SSB consumption remains high and provides an overview of the variations in consumption based on WHO regions, country income levels, and age groups. On average, children worldwide consume 326 mL/day of SSB; however, this varies from 115 mL/day in Australia to 710 mL/day in China [36].

The mean±SD DMFT in this study was 4.58±2.73, and the DMFS was 7.63±4.52. Just 7% of the individuals were caries-free, while the great majority 93% had at least one tooth with dental caries. The findings were comparable to those of a study carried out in Karbala city, Iraq, which revealed that 95.60% of 500 male students, all aged 15 years, had dental caries, with an average DMFT of 6.396±0.157 [37]. The region's high dental caries prevalence necessitates collaboration with local health authorities, dental professionals, and community organizations to develop effective strategies to reduce sugar-sweetened beverage consumption.

The main finding of this study was a significant association between SSB consumption and DMFT following adjustment of potential confounders. A recent systematic review of observational studies showed that when comparing those with moderate-to-low intakes, low consumption (never/low SSB consumption <71 mL/day, <2 times/week), and moderate SSB consumption (71-250 mL/day, 2-7 times per week), the risk of dental caries raised significantly (OR=1.57, 95% CI: 1.28-1.92). DMFT weighted mean differences (WMD) = 0.82, 95% CI: 0.38-1.26; while comparing high-to-moderate intakes, moderate SSB consumption (71-250 mL/day, 2-7 times per week), high SSB consumption (>250 mL/day, >1 time/day), the dental caries risk elevated more (OR=1.53, 95% CI: 1.17-1.99, DMFT WMD=1.16, 95% CI: -0.59-2.91). A clear and consistent relationship between the dosage of exposure and the occurrence of caries was identified, indicating a dose-response gradient [6]. An Iranian study, which recruited a total sample of 600 teenagers, showed that the mean DMFT index of the subjects was 4.0±3.0. The DMFT score for teenagers who never consumed sweetened soft drinks was 39% lower than that of those who regularly consumed them. However, the DMFT score of teenagers who drank milk on a daily basis was significantly lower than that of those who had not drunk milk in the previous three months [26]. Compared to sucrose, glucose, and fructose, milk sugar (lactose) induces a lesser plaque pH reduction [38]. In the current study, we solely assessed the frequency of SSBs consumption without investigating the specific patterns of consumption. However, it is noteworthy that the consumption of milk in this particular population was very low, with only 8% of individuals reporting milk consumption (plain milk). Out of this number, only four students reported drinking their milk without adding sugar.

On the contrary, Pitchika et al. examined the possible links between the intake of SSB and dental caries among children from two German birth cohorts who were monitored at the ages of 10 and 15 years [30]. A significant relationship between the intake of SSB and the caries experience was detected during the 10-year follow-up period, as measured by decayed, missing, filled surfaces (DMF/S); non-cavitated carious lesion (NCCL/S); and (overall caries burden) DMF+NCCL/S criteria. Nevertheless, during the 15-year follow-up, a diminished correlation between SSB intake and caries was observed. Studies suggest that despite increased consumption of SSB in industrialized countries, dental caries have decreased. This decline may be attributed to the use of preventative treatments such as the use of fluorides and other methods [39]. On the other hand, the prevalence of caries is still rather high in a number of developed countries [40]. Fluorides may have raised the level at which sugar intake is considered acceptable to a certain degree [41]. Factors such as positive sampling, the "Hawthorne effect," and participants providing inaccurate information about their SSB consumption were among the factors that might have contributed to the attenuated effect [30]. Likewise, another study found no correlation between self-reported SSB use and dental caries among Alaska Natives. The small sample size (n=51) may have contributed to the null results of this cross-sectional investigation [42].

Ensuring comprehensive dietary data collection by using 24-hour recalls on two occasions, including a weekend day, allows for measuring total sugar intake, SSB sugar content, and total caloric intake. In addition to covering a wide range of other confounding variables, a good sample size added to the strength of the study.

However, the study had several limitations. Despite the appropriate sample size, the findings may not be representative of the entire population of Iraq, as drink and food habits may vary from one area to another due to cultural and environmental factors. Moreover, the study did not consider factors, such as whether SSB consumption occurred with or between meals, despite efforts to control confounding factors. Self-reported information, such as beverage and dietary intakes and oral health-related habits, might lead to inaccurate data due to recall bias and social desirability biases. Students may provide socially acceptable responses and they may underreport their SSB consumption or overstate their adherence to oral-health-related habits to avoid judgment or criticism. Participants may have difficulty accurately remembering all the foods and beverages they consumed over the recall period (the past 24-hour period). Memory declines and forgetfulness can lead to underreporting or incomplete reporting of dietary and beverage intakes. Furthermore, this study looked at the frequency of sugar consumption on a daily basis only, without considering the effects of different types of SSBs on dental caries. The cross-sectional nature of this study did not allow for the interpretation of causal associations, and seasonal variation in food and beverage intakes could not be assessed.

Studies found that a lack of knowledge regarding the composition of SSB beverages may lead to increased SSB consumption [43]. Future studies should investigate adolescents' knowledge, attitudes, and awareness about SSB contents and the risks they pose to their oral and general health. In addition, future research assessing the pattern of consumption of each type of SSB and its effects on dental caries in the Kurdistan region is recommended.

## Conclusions

This study showed considerably high consumption levels of sugar-sweetened beverages among adolescents in Erbil, Iraq. The consumption of sugar-sweetened beverages was significantly higher among male students compared to female students. Adolescents in Erbil experienced a very high dental caries rate. Following adjustment of confounding variables, there was a significant association between dental caries experience and frequency of sugar-sweetened beverage consumption. Hence, public health efforts should emphasize reducing the intake of these beverages as part of broader strategies to promote oral and general health. Education campaigns, policy interventions, such as taxation and restrictions on marketing to children and adolescents, and community-based programs aiming to decrease sugar-sweetened beverage consumption, are strongly recommended.
