# Correction: Sugar-Sweetened Beverage Consumption and Its Association With Dental Caries Among Adolescents in Erbil, Iraq: A Cross-Sectional Study

**DOI:** 10.7759/cureus.c186

**Published:** 2024-07-18

**Authors:** Heran I Hassan, Samir M Othman

**Affiliations:** 1 Orthodontics, Pediatric and Preventive Dentistry, College of Dentistry, Hawler Medical University, Erbil, IRQ; 2 Community Medicine, College of Medicine, Hawler Medical University, Erbil, IRQ

This article has been corrected due to an error in the abstract. The second paragraph of the abstract originally included the following: “tooth (DMFT) was 94.58 ± 2.73.” This has been corrected to: “tooth (DMFT) was 4.58 ± 2.73.” The authors and journal deeply regret that this typo was not identified and addressed prior to publication.

